# RBM10 modulation of circRNA biogenesis contributes to its tumor suppressor role in lung adenocarcinoma

**DOI:** 10.1186/s40364-026-00891-6

**Published:** 2026-02-12

**Authors:** Ana Utrilla-Maestre, Ana M. Matia-González, Paola Peinado, Maria Angeles Becerra-Rodriguez, Maria S. Benitez-Cantos, Marta Cuadros, Pedro P. Medina

**Affiliations:** 1 Department of Biochemistry and Molecular Biology I, Faculty of Sciences, University of Granada, Granada, Spain; 2 GENYO. Centre for Genomics and Oncological Research Pfizer, University of Granada, Andalusian Regional Government, Granada, Spain; 3 Instituto de Investigación Biosanitaria de Granada (ibs.GRANADA), Granada, Spain; 4 Present Address: The Francis Crick Institute, London, UK; 5 Department of Biochemistry and Molecular Biology III, Faculty of Medicine, University of Granada, Granada, Spain

**Keywords:** RBPs, LUAD, Back-splicing, circSMARCA5, circHIPK3, Circular RNA

## Abstract

Circular RNAs (circRNAs) are emerging regulators in cancer biology, yet the mechanisms underlying their biogenesis remain incompletely defined. RBM10, a splicing regulator frequently mutated in lung adenocarcinoma (LUAD), modulates RNA processing, but its involvement in circRNA regulation has not yet been addressed. Transcriptomic profiling of RBM10-restored LUAD cells, followed by RT-qPCR validation, identified circHIPK3 and circSMARCA5 as consistently RBM10-dependent circRNAs. Subcellular fractionation confirmed nuclear confinement of RBM10 and cytoplasmic enrichment of circRNAs, supporting a nuclear role for RBM10 in circRNA biogenesis. PAR-CLIP and RNA pulldown assays demonstrated direct RBM10 binding to intronic flanking regions of these circRNAs. Using a splicing reporter assay, we found that RBM10 binding to the 3' flanking region promotes exon skipping and circularization more efficiently than 5' binding, revealing a position-dependent mechanism controlling circRNA output. Analysis of RBM10 point mutants showed impaired regulation of circHIPK3 and circSMARCA5, linking defective exon skipping to disrupted circRNA formation. Functionally, modulation of circHIPK3 and circSMARCA5 phenocopied RBM10 restoration in mutant LUAD cell lines and rescued the tumorigenic phenotype driven by RBM10 loss. In two independent LUAD cohorts, circHIPK3 was consistently downregulated, particularly in RBM10-mutant tumors, and strongly correlated with RBM10 expression. Proteomic analyses further identified RBM10–SF3B1 interaction as a key upstream event governing circHIPK3 biogenesis. Together, these findings uncover a previously unrecognized mechanism through which RBM10 exerts tumor-suppressive functions via circRNA regulation and highlight circHIPK3 as a promising biomarker and potential therapeutic target in RBM10-deficient LUAD.

**Supplementary Information:**

The online version contains supplementary material available at 10.1186/s40364-026-00891-6.

## Graphical Abstract

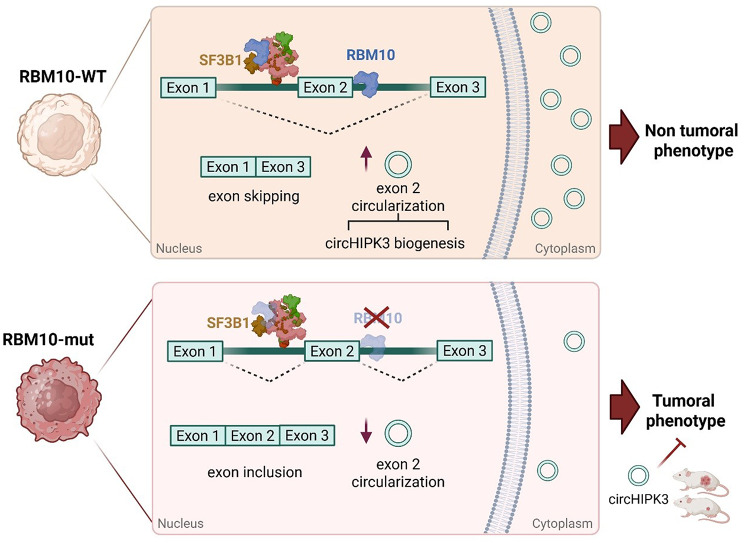

To the editor,

Lung adenocarcinoma (LUAD) remains the leading cause of cancer mortality, requiring deeper molecular insight to improve diagnosis and treatment. The RNA-binding motif protein 10 (RBM10) is a LUAD driver gene [1], mutated in ~ 7% of cases [2], often through loss-of-function (LOF) mutations that compromise its tumor-suppressive role [3]. RBM10 is an RNA-binding protein (RBP) involved in alternative splicing [4]. Likewise other RBPs, the role of RBM10 could go beyond mRNAs and regulate the expression of circular RNAs (circRNAs) either enhancing or inhibiting their formation [5, 6]. CircRNAs are stable non-coding RNAs produced by back-splicing, often facilitated by Alu-rich introns [7], and they influence gene regulation by acting mostly as microRNA sponges. CircRNA dysregulation is widely reported in cancer [8].

To explore RBM10’s potential role in circRNA biogenesis, we performed total RNA-sequencing after restoring of the predominant RBM10 isoform 2 (Fig.S1A) in the RBM10-LOF-mutant NCIH1944 cell line (Fig. S1B). From the 656 consistently detected circRNAs, 99 were downregulated (FC _RBM10/EV_ ≤ 0.80) and 79 upregulated (FC _RBM10/EV_ ≥1.2) in both replicates (Fig. 1A, 1B; File S1). For RT-qPCR validation, we prioritized circRNAs with higher read counts (Table S1) including circRNF13, circSMARCA5 and circTNFRSF21 (downregulated) and circCYP24A1, circHIPK3, and circSPECC1 (upregulated). Except for circRNF13, whose amplification could not be achieved, the specificity of circRNA detection was validated using convergent and divergent primers together with RNase R digestion (Fig. S2A, S2B). RBM10-dependent expression of circSMARCA5, circCYP24A1 and circHIPK3 was further confirmed by RT-qPCR in two independent RBM10 restoration models (NCIH1944 and NCIH2291) and in a knockdown model of RBM10 (NCIH23) (Fig. 1C).

Cellular fractionation revealed that RBM10 localizes exclusively in the nucleus, while circRNAs are mainly cytoplasmic (Fig. 1D), supporting a role for RBM10 in nuclear circRNA biogenesis rather than downstream cytoplasmic processes. Analysis of PAR-CLIP data from the HEK293T cell line [9] revealed enriched RBM10–RNA crosslinking at the 3' flanking site (FS) of circHIPK3 and the 5' FS of circSMARCA5 (Fig. 1E). Due to the lack of reads mapped to the FS of circCYP24A1, no RBM10-circCYP24A1 interaction could be confirmed suggesting an indirect regulation (Fig. S3A). RNA pulldown assays using biotinylated transcripts incubated with NCIH1944-RBM10 extracts confirmed direct interaction of RBM10 to these regions in LUAD cells, guaranteed by the addition of a cold RNA competitor (Fig. 1F; Fig S3B).

Exon skipping has been correlated with circularization [10] so, to mechanistically link RBM10 binding position to circRNA output, we employed a splicing reporter system [11] to recruit RBM10 to either the 5' or 3' FS and assess its impact on splicing (Fig. 1G). RBM10 binding to the 3' FS promoted exon skipping more effectively than binding to the 5' FS, with an approximately 3-fold increase. Our findings suggest that RBM10 may facilitate circularization through the promotion of exon skipping.

To ensure that direct RNA binding is required for RBM10-dependent circularization, we analysed two well-characterized mutants in RNA-binding domains: R343G (RRM2) and S781L (ZnF2) (Fig. 1H). In NCIH1944 cells, overexpression of either mutant led to a significant impairment in circHIPK3 and circSMARCA5 compared to wild-type (WT) RBM10, indicating that these point mutations affect RBM10-dependent circRNAs regulation. Additionally, the splicing reporter showed that R343G severely disrupts exon skipping, reinforcing the mechanistic notion that RBM10-driven exon skipping constitutes a critical upstream event that may subsequently facilitate circRNA formation.

**Fig. 1:**
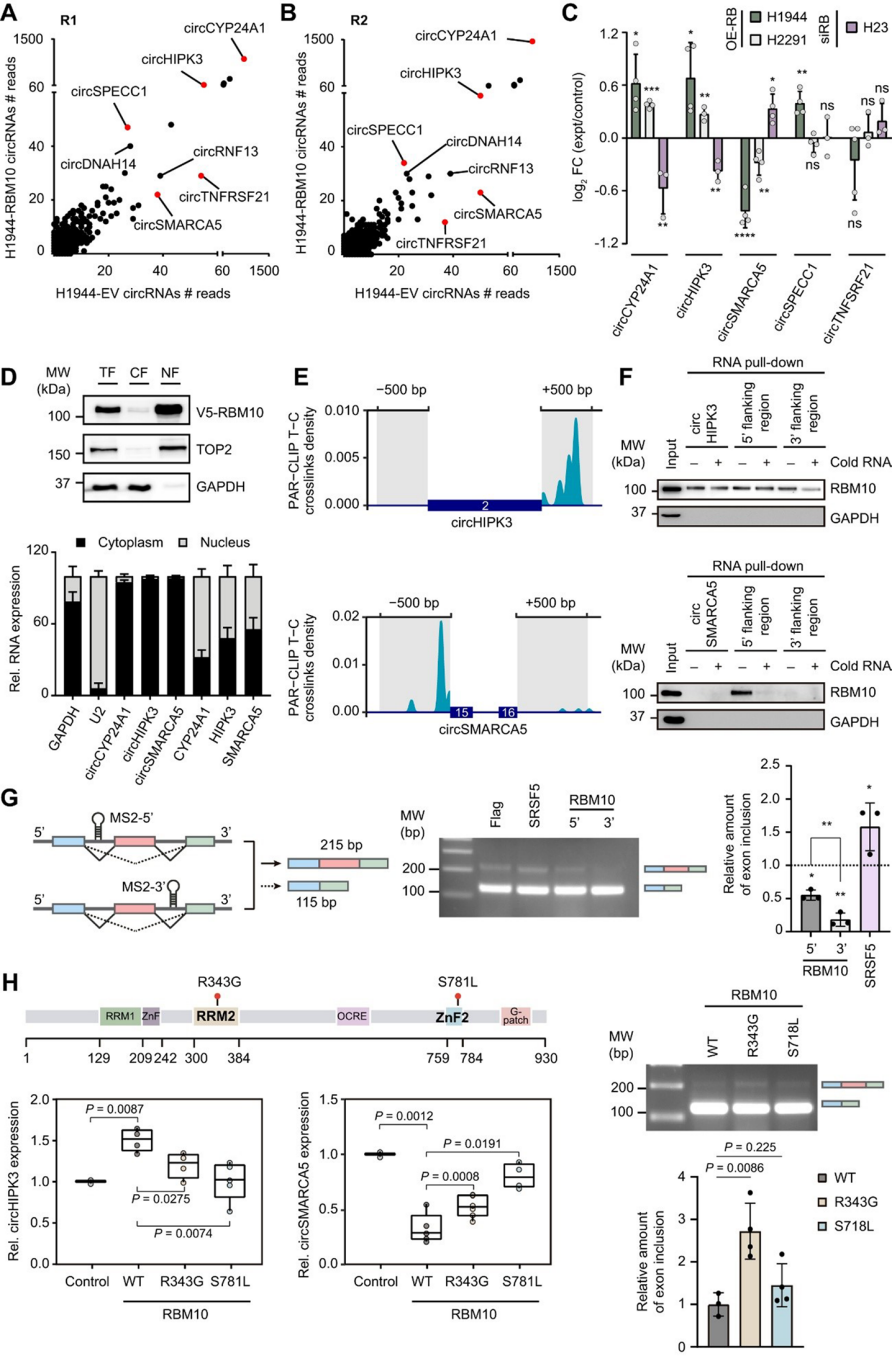
Scatter plot depicting the number of reads of circRNAs identified in the first (**A**) and second (**B**) biological replicates of NCIH1944 cell line transfected with EV or RBM10. Red dots indicate circRNA selected for further validation. (**C**) RT-qPCR validation showing the log_2_ Fold Change of circRNA expression upon RBM10 restoration in NCIH1944 (dark green) and NCIH2291 (light green), or RBM10 silencing in NCIH23 (purple). Baseline (0.0) represents control condition. Error bars indicate SEM (* *P* < 0.05, ** *P* < 0.01, *** *P* < 0.001, **** *P* < 0.0001, ns: not significant, unpaired t-test). (**D**) Subcellular fractionation showing RBM10 protein (Western blot, top) and circRNA distribution (RT-qPCR, bottom) in total fraction (TF), cytoplasmic fraction (CF), and nuclear fraction (NF). TOP2 and GAPDH serve as nuclear and cytoplasmic markers. (**E**) RBM10 PAR-CLIP binding sites in circHIPK3 and circSMARCA5 flanking regions. T-to-C transitions indicate crosslinking sites and grey boxes represent the regions analyzed in the pull-down experiment. (**F**) RNA–protein complexes formed between biotin-labeled circHIPK3 or circSMARCA5 and their 5'/3' flanking sites and extracts of NCIH1944 expressing RBM10. A cold RNA competitor was added to assess the specificity of the interaction. (**G**) Scheme of splicing reporters and resulting isoforms after cellular mRNA processing (left). Agarose gel showing splicing of the *lucMAPT-30D* (*3'*) reporter following co-transfection with MCP-RBM10, SRSF5 (positive control) or Flag (negative control). In lane 3, *lucMAPT-30U* (*5'*) was co-transfected with MCP-RBM10 (centre). Relative quantification of exon inclusion normalised to Flag negative control, marked as a dashed line (right). (**H**) RBM10 protein structure showing R343G (RRM2) and S781L (ZnF2) mutation locations. (Upper left). CircHIPK3 and circSMARCA5 expression in wild-type and mutants RBM10, normalised with the parental control (Bottom left). Agarose gel showing a splicing reporter co-transfected with RBM10-WT, R343G, or S781L mutants and relative quantification of exon inclusion normalized to RBM10-WT (Right). *P*-values have been calculated with an unpaired t-test

To assess the functional relevance of RBM10-regulated circRNAs, we overexpressed circHIPK3 and silenced circSMARCA5 in LUAD cell lines. These transfections effectively modulated circRNA levels without altering their corresponding linear transcripts (Fig. S4). Both circHIPK3 overexpression in NCIH1944 (Fig. 2A) and NCIH2291 (Fig. S5A) cells and circSMARCA5 knockdown (Fig. S5B) significantly decreased colony formation and cell viability, mimicking the effects of RBM10. Importantly, the tumorigenic phenotype promoted by the loss of RBM10 in NCIH23 WT LUAD cells could be functionally rescued by either overexpressing circHIPK3 (Fig. 2B) or silencing circSMARCA5 (Fig. S5C), suggesting that these circRNAs manipulation represents a potential therapeutic strategy specifically for RBM10-mutant lung tumors. circHIPK3 overexpression reduced xenograft tumor weight (Fig. 2C) by over 50%, confirming its tumor-suppressive role in vivo. However, circSMARCA5 silencing did not yield significant in vivo effects (Fig. S5D).

To enhance the translational relevance of our findings, we assessed circHIPK3 and circSMARCA5 expression in LUAD patients. CPTAC cohort showed that both circRNAs were significantly reduced in tumoral samples (Fig. 2D, left; Fig. S6). The decrease in circHIPK3 is consistent with our model in which RBM10 loss suppresses its expression and contributes to tumor progression. In contrast, circSMARCA5 downregulation does not align with its oncogene-like behavior observed in vitro, suggesting that although circSMARCA5 is mechanistically influenced by RBM10, its clinical relevance in LUAD may be limited or restricted to specific RBM10-deficient contexts. Thus, we focused subsequent clinical analyses on circHIPK3, whose robust tumor-suppressive profile in patients provides a clearer translational direction. To validate these findings on circHIPK3, we analyzed our in-house LUAD cohort. From 70 patients, a representative set of 13 paired tumoral and adjacent non-tumoral samples was examined. Consistent with CPTAC, circHIPK3 levels were significantly reduced in tumors (Fig. 2D, right), reinforcing the robustness of our observations.

We next examined the relationship between circHIPK3 and RBM10 in our in-house cohort, as the public dataset lacked sufficient RBM10-deficient cases. circHIPK3 expression strongly correlated with RBM10, whereas no correlation was observed with the linear mRNA (Fig. 2E). Consistently, RBM10-mutant tumors showed even greater circHIPK3 downregulation (Fig. 2F), further supporting RBM10-dependent regulation in the tumoral context.

Finally, to test whether RBM10 engages with the core spliceosome to regulate circRNAs, we performed coimmunoprecipitation (Co-IP) followed by mass spectrometry (MS) assays in NCIH1944-RBM10 cells (Fig. S7A). Label-free quantification analysis revealed strong enrichment of U2 complex proteins (Fig. 2G), being SF3B1, SF3B2 and DHX15 further confirmed by Co-IP (Fig. S7B). Several recent studies demonstrate that SF3B1 mutations can alter back-splicing decisions, giving rise to deregulated circRNAs [12]. Notably, SF3B1 silencing resulted in significant upregulation of circHIPK3 (Fig. 2H) and RBM10 restoration in SF3B1-silenced cells failed to further increase circHIPK3 levels (Fig. 2I), demonstrating that SF3B1 is epistatic to RBM10 in this regulatory pathway. Hence, these results indicate that the RBM10-SF3B1 axis specifically regulates circHIPK3 formation.

**Fig. 2:**
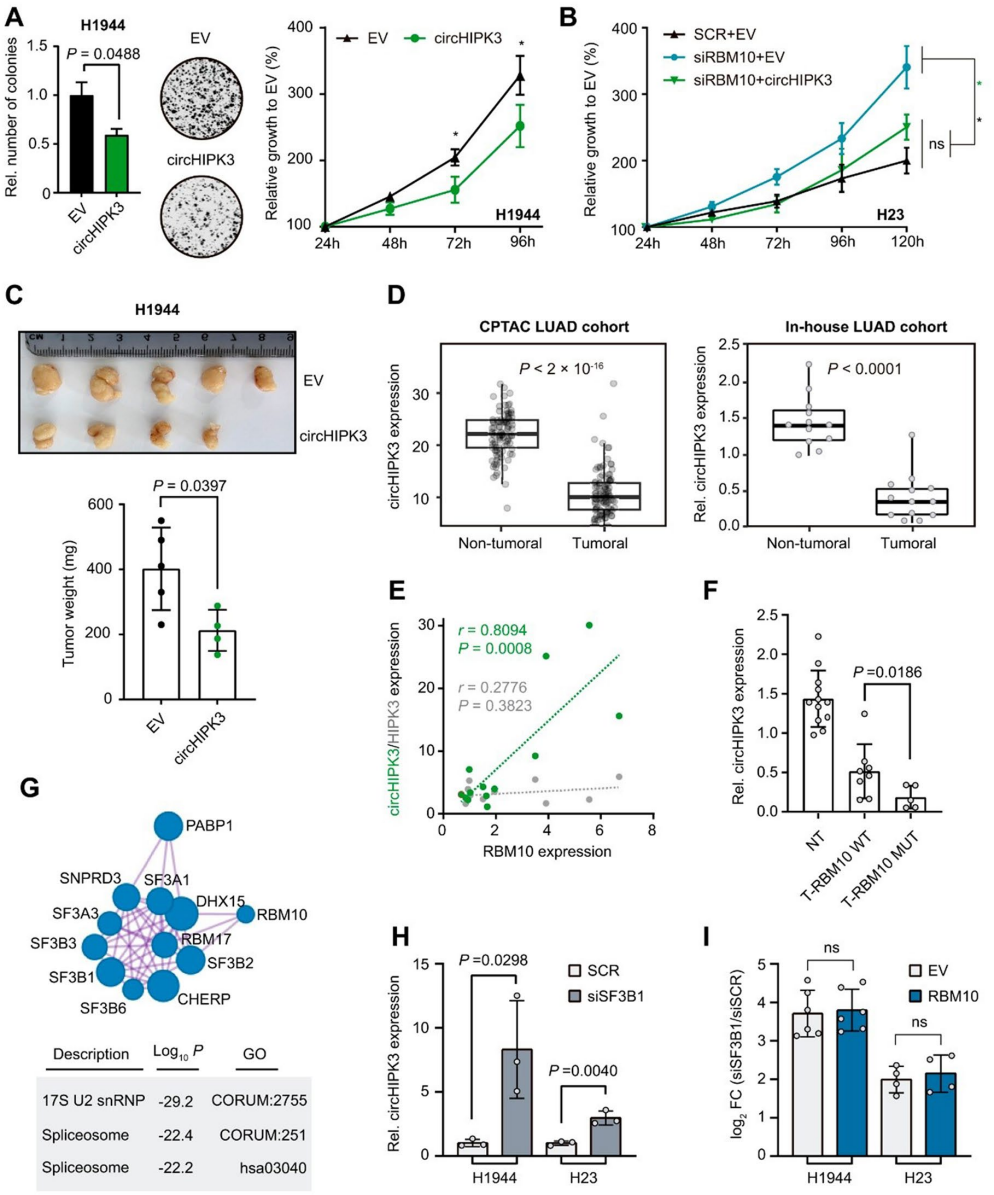
(**A**) Clonogenic and viability assays after circHIPK3 ectopic expression in NCIH1944 (* *P* < 0.05, unpaired t-test). (**B**) Rescue experiment in NCIH23 WT cells under RBM10 silencing and subsequent modulation of circHIPK3 (* *P* > 0.05; ns: non- significant; unpaired t-test at 120 h). (**C**) Image and weight’s quantification of ex vivo tumors of NCIH1944 circHIPK3 or EV cell-derived xenografts. (**D**) Box plot comparing circHIPK3 expression between non-tumoral and tumoral samples from the CPTAC and in-house LUAD cohorts (Mann-Whitney test). (**E**) Scatter plot showing the correlation between RBM10 expression and either circHIPK3 (green dots) or HIPK3 (grey dots) from the in-house LUAD patient cohort. Pearson correlation (*r*) and *P* values (*P*) are indicated. (**F**) Relative circHIPK3 expression across Non-Tumoral (NT) samples, tumoral samples with wild-type RBM10 (T-RBM10 WT) or RBM10 mutated (T-RBM10 MUT) from the in-house LUAD patient cohort (Mann-Whitney test). (**G**) Immunoblot monitoring the immunoprecipitation of V5-RBM10 using an anti-V5 antibody, and IgG as control. GAPDH was used as a negative control for non-specific binding. (**H**) Mass Spectrometry (MS) analysis. Left: Network visualization of proteins involved in splicing identified as significantly enriched in the V5-RBM10 IP. Right: Table showing selected enriched Gene Ontology (GO) terms, their functional descriptions and log_10_ *P*-values. (**I**) circHIPK3 expression following the silencing of SF3B1 (siSF3B1) in two LUAD cell lines (NCIH1944 and NCIH23), relative to a scrambled siRNA control (siSCR) (unpaired t-test). (**J**) Log_2_ Fold Change (log_2_FC) expression for circHIPK3 following the silencing of SF3B1 (siSF3B1) in NCIH1944 and NCIH23 relative to a scrambled siRNA control (siSCR). RBM10 was restored after silencing, using an empty vector as control. GAPDH was used as an endogenous control (ns = not significant, unpaired t-test)

Our findings demonstrate that RBM10 exerts its tumor-suppressive role by directly regulating circRNA biogenesis through its binding to their intronic flanking regions. Its position-dependent binding determines the outcome: downstream binding promotes exon skipping and circularization (e.g., circHIPK3), whereas upstream binding inhibits it (e.g., circSMARCA5). To accomplish this, RBM10 engages with the core spliceosome, with a particular reliance on SF3B1. Among circRNAs regulated by RBM10, clinical data support circHIPK3 as a promising candidate for further investigation as RNA-based biomarkers or therapeutic tools. 

## Supplementary Information

Below is the link to the electronic supplementary material.

Supplementary Material 1

Supplementary Material 2

Supplementary Material 3

Supplementary Material 4

## Data Availability

Total RNA-seq data of H1944 cell line is available in Gene Expression Omnibus database under the accession ID GSE000000. Mass spectrometry data is available in ProteomeXchange under the accession ID PXD071457.

## Abbreviations

LUAD: Lung adenocarcinoma
LOF: Loss of function
circRNA: Circular RNA
RBM10: RNA-binding motif protein 10
RBP: RNA-binding protein
PAR-CLIP: Photoactivable-Ribonucleoside-Enhanced crosslinking and Immunoprecipitation
RNase R: Ribonuclease R
RT-qPCR: Reverse transcription quantitative PCR
FC: Fold Change
FS: Flanking site
Co-IP: Coinmunoprecipitation
MS: Mass spectrometry
