# Supplementary material for: RBM10 modulation of circRNA biogenesis contributes to its tumor suppressor role in lung adenocarcinoma
Source: Biomark Res. 2026 Feb 12;14:24. doi: 10.1186/s40364-026-00891-6 (PMC12895834; doi:10.1186/s40364-026-00891-6)
Supplement: Supplementary file 4 — Supplementary Material 4 [file 40364_2026_891_MOESM4_ESM.docx]

**RBM10 modulation of circRNA biogenesis contributes to its tumor suppressor role in lung adenocarcinoma.**

Utrilla-Maestre *et al*

# Table of contents

[Table of contents 1](#_Toc215575103)

[Methods 2](#_Toc215575104)

[Extended Data Figures 12](#_Toc215575105)

[Extended Data Tables 23](#_Toc215575106)

[Supplementary File Descriptions 25](#_Toc215575107)

[References 26](#_Toc215575108)

#

# Methods

Cell culture

Lung adenocarcinoma cell lines (NCIH1944, NCIH2291, NCIH23, NCIH1435, NCIH1437, NCIH1975, NCIH2009, A427 and A549) were acquired from the ATCC collection and mycoplasma contamination testing was conducted. They were maintained in Roswell Park Memorial Institute (RPMI) medium with 10% fetal bovine serum (FBS), L-glutamine, antibiotics and antimycotic agents (1% penicillin/streptomycin/amphotericin) at 37 ºC in an atmosphere of humidified air with 5% of carbon dioxide. Normal bronchial epithelial cells, NL20, were grown under standard culture conditions (37 ºC, 5% carbon dioxide) in Ham’s F12 medium with 4% FBS, 2.0 mM L-glutamine, 1.5 g/L sodium bicarbonate, 2.7 g/L glucose, 0.1 mM nonessential amino acids, 1 μg/mL transferrin, 5 μg/mL insulin, 10 ng/ml EGF, and 500 ng/mL hydrocortisone.

Analysis of RBM10 isoforms

Total RNA was isolated from NL20 cells with TRI Reagent (Sigma). RBM10 transcripts were amplified by PCR and the products were gel-purified and ligated into the pGEM-T Easy vector (Promega). The ligation product was used to transform *Escherichia coli* DH5α cells following manufacturer specifications. Positive colonies were screened by performing a restriction digest assay with EcoRI and further validated by Sanger sequencing.

To analyze the expression of RBM10 isoforms *in silico* in external datasets, we downloaded RNA-Seq BAM files from Gillette et al [1] via dbGaP (phs001287) (111 tumor and 102 normal samples). We also used GEPIA2 [2] to explore isoform expression in TCGA-LUAD. For cell lines, we used our own RNA-Seq data from NCIH1944 and NCIH1975 as well as in-house RNA-Seq data from A549 (unpublished data). We used RefSeq for isoform annotation: NM_005676 (Isoform 1), NM_152856 (Isoform 2), NM_001204466 (Isoform 3), NM_001204467 (Isoform 4) and NM_001204468 (Isoform 5). To quantify the relative use of local splice junctions, we used MAJIQ [3] and then we inferred isoform use based on local splice junction use.

Transfections

For RBM10 restoration, a plasmid containing the isoform 2 of RBM10 was constructed (pcDNA4-iso2RBM10), using pcDNA4-Neo as a backbone. Specifically, the coding sequence of the RBM10 isoform 2 was subcloned from the RBM10_pLX307 plasmid (gift from William Hahn & Sefi Rosenbluh [4]; Addgene plasmid #98371; <http://n2t.net/addgene:98371>; RRID:Addgene_98371). 3 million NCIH1944 or 2.5 million NCIH2291 cells were seeded in p100 dishes and transfected with 5 µg of the pcDNA4-iso2RBM10. For circRNAs ectopic expression, candidate circRNAs (circHIPK3: hsa_circ_0000284; circCYP24A1: hsa_circ_0060927) were cloned into the pcDNA3.1 (+) circRNA Mini Vector backbone (gift from Jeremy Wilusz [5]; Addgene plasmid #60648; <http://n2t.net/addgene:60648>; RRID:Addgene_60648). 750,000 NCIH1944 or 500,000 NCIH2291 cells were seeded in six-well plates and transfected 24h later with 1 µg of the corresponding plasmid. In all the cases, the empty-vector (EV) was used as control and transient transfections were performed with *Trans*IT®-LT1 Transfection Reagent (Mirus) in OptiMEM medium following manufacturers’ recommendations.

For circSMARCA5 silencing, 60-80% confluent cells were transfected with 40 nM of a silencing RNA (siRNA) against the junction sequence of the desired circRNA. The si-circSMARCA5 [6] was transfected using Lipofectamine RNAiMAX reagent (Invitrogen MA USA) according to the manufacturer's protocol. As control, scramble siRNA (siSCR; Ambion, #4390843) was used. Same protocol was used for RBM10 and SF3B1 silencing, using the following siRNAs: siSF3B1#1: 5'-GCATAGGCGGACCATGATA-3’; siSF3B1#2: 5'-CCGGGAAGATGAATACAAA-3' [7] and ON-TARGETplus Human RBM10 (8241) siRNA- SMARTpool (Dharmacon).

Total RNA sequencing and circRNA analysis

Cell line NCIH1944 was subject to transfection with pcDNA4-iso2RBM10 and backbone plasmid as control in biological duplicates. After 48 hours, total RNA extraction was carried out using mirVana™ miRNA Isolation Kit, with phenol (Cat#AM1560, Thermo Fisher Scientific) according to manufacturer's instructions. RNA samples were sent to CNAG-CRG Genomic Unit (Barcelona, Spain) for sequencing. Briefly, libraries were prepared using TruSeq Stranded Total RNA Library Prep Kit with Ribo-Zero Human/Mouse/Rat Kit (REF. RS-122-2201/2202, Illumina) and sequenced with Illumina HiSeq 2500 system generating paired-end 125 bp-long reads.

Quality control of the raw FASTQ files was performed using FastQC (v0.11.8). Reads were aligned to the human genome GRCh38.d1.vd1. using STAR (v2.6.1b) in two-pass mode. To allow for chimeric reads to be detected, --chimSegmentMin parameter was set to 10. Then, CIRCexplorer2 tool (v2.3.8 [8]) was used to perform annotation-free detection and quantification of circRNAs. From the total list of circRNAs identified, those circRNAs absent in at least one replicate were discarded for downstream analyses.

Genomic DNA (gDNA) / RNA / protein extraction

gDNA was extracted using QuickExtract™ DNA Extraction Solution (Biosearch Technologies) and total RNA for real-time PCR was purified using TRI Reagent (Sigma) following the manufacturer’s guidelines. Total protein was extracted by incubating the samples with RIPA extraction and lysis buffer (Thermo Fisher Scientific) supplemented with a phosphatase and protease inhibitor cocktail on ice for 20 min. Lysate was obtained after centrifugation at 14,000 ×*g*, at 4 ºC for 15 min and removal of cell debris.

RNAse R treatment

Purified RNA from the NCIH1944 cell line was incubated at 37 ºC for 2 min with Ribonuclease R (RNase R) according to Biosearch Technologies recommendations. Samples were consecutively incubated at 65 ºC for 20 minutes for the inactivation of the enzyme.

Reverse transcription quantitative PCR (RT-qPCR)

2 μg of RNA was treated with DNaseI (Invitrogen) and reverse transcriptase (RevertAid RT kit, Thermo Fisher Scientific) to remove contaminant DNA and synthesize complementary DNA (cDNA), respectively. Then, cDNAs were used as template for a SYBR Green quantitative PCR reaction to quantify gene expression at RNA level using the KAPA SYBR®FAST (Merck) and the QuantStudio™3 Real-Time PCR System (Thermo Fisher Scientific). At least three technical replicates were performed for each biological replicate. The ΔΔCt method was used to calculate relative expression normalizing against an endogenous control. The list of oligonucleotides is included in supplementary material (**File S2**).

Subcellular fractionation

Total, nuclear and cytoplasmic fractions were separated from NCIH1944 cell pellets following the cell fractionation protocol optimized by Beringer et al, 2016 [9]. 10% and 60% of each fraction was used to monitor the localization of the desired proteins by immunoblot or RNAs by qPCR, respectively.

*In vitro* transcription (IVT)

Specific oligonucleotides were designed to amplify candidate circRNAs and their flanking regions (~600 nt) from NCIH1944 cDNA or DNA, respectively (Table 2). The T7 promoter sequence (TAATACGACTCACTATACCCCAACC) was included in the 5’ region of each oligonucleotide. In-gel purified amplicons were further incubated with T7 RNA polymerase (Promega) and biotinylated (Biotin RNA labeling mix, Roche) or non-biotinylated nucleotides for 4 hours at 37 ºC to obtain the corresponding RNA. Then, samples were treated with DNaseI (Invitrogen) to eliminate DNA templates and purified with RNeasy kit (Qiagen).

Biotinylated RNA pulldown

Total protein was extracted from NCIH1944 cells 48h after the transfection with pcDNA4-iso2RBM10. 100 μg of the protein was incubated (30 min, 850 rpm, 25 ºC) with 10 pmol of the biotinylated RNA, DNaseI and RNase OUT (Invitrogen). Then, each sample was incubated (30 min, 25 ºC, 950 rpm) with 50 μL of Dynabeads™ M-280 Streptavidin (10 mg/mL; Thermo Fisher Scientific). The beads were washed with IP buffer (20mM Tris, pH: 7,5; 150 nM NaCl; 1mM EDTA; 5% glycerol; 0,5% triton; 1mM DTT, 0,1 mg/mL heparin; 0,1 mg/mL tRNA; protein inhibitors) for 4 times. Finally, RBM10 was monitored in the eluates by immunoblot. A 3-fold excess of the corresponding non-biotinylated RNA was added as a cold competitor in each experiment to ensure the specific nature of the interaction.

Rescue experiments

For rescue experiments, RBM10-wildtype cell line NCIH23 was used. 500,000 cells were seeded in p6 plates and after an overnight, we treated the cells with siRNA-RBM10 or siSCR as control. The next day, circRNA modulation was performed overexpressing circHIPK3 or silencing SMARCA5. Detailed protocol can be found in the previous section “Transfections”. Cell proliferation was monitored during the next 6 days.

RBM10 mutant’s experiments

To test how different mutations on RBM10 affects to circRNAs expression, we transfected mutant NCIH1944 cells with different plasmids provided by Valcarcel group expressing RBM10 WT (pcw57.1 RBM10 WT FLAG), RBM10 S781L (pcw57.1 RBM10 S781L) and RBM10 R343G (pc57.1 RBM10 R343G), mutations affecting the ZnF2 and RRM2 of RBM10, respectively. A plasmid encoding RBM10 WT with a mutation in the initiation of translation codon was used as negative control. Cells were transfected in p6 plates with 1 μg of plasmids and 24h later, the expression of the different RBM10 versions was induced with doxycycline for 3 days. Cells were collected, RNA was extracted and retrotranscribed. RBM10 induction was check by qPCR and circRNAs expression was measured by qPCR in each condition. Expression was normalized to the negative control.

Minigene experiment

To investigate the effect of RBM10 binding location on a splicing reporter, we used a previously described minigene construct containing exons 9 to 11 of *MAPT*, with exon 10 flanked by 500 base pairs of its native intronic sequence [10]. MS2 stem-loop sequences were inserted either 30 nucleotides upstream (*lucMAPT-30U*) or 30 nucleotides downstream (*lucMAPT-30D*) of exon 10 to enable site-specific recruitment of MCP-fused RBM10 proteins. These reporters were co-transfected with plasmids expressing MCP-RBM10 fusion protein, allowing RBM10 to be tethered to defined positions within the pre-mRNA. A plasmid encoding SRSF5 was used as a positive control as it is known to promote exon inclusion when it binds to the downstream region while an empty vector with the same backbone (FLAG) was used as negative control.

7.5 μg of splicing reporter (either lucMAPT-30U or lucMAPT-30D) was cotransfected with 7.5 μg of MCP-RBM10, SRSF5 or FLAG into 3 millions of NCI-H1944 cells in p-100 plates. After 48 hours, RNA was isolated and RT‐PCR was performed using the following pair of primers: lucMAPT-Fw (AGAACCTGAAGCACCAGCC) and lucMAPT-Rv (acaccttggaagcTGGTTTATGATGGATGTTGCCTAATGAG). Amplicons were run into a 3% agarose gel for visualization and bands were quantified by ImageJ normalizing to FLAG.

Western blot

Total protein amount was quantified following the Bradford method [11]. 25 to 40 µg of the lysate was loaded and resolved in a SDS-PAGE electrophoresis gel and transferred to PVDF membranes. Membranes were first blocked in 5% of non-fat dry milk in T-PBS for an hour at room temperature (RT), then incubated overnight with specific primary antibodies at 4 ºC and finally incubated for 90 min at RT with the corresponding secondary antibodies (**Table S2**).

The protein bands were visualized using the *SuperSignal West Femto Maximum Sensitivity Substrate* (Thermo Scientific) and ImageQuant LAS4000 (GE Healthcare, Chicago, IL, USA). Bands intensity was quantified using the Plot Lanes tool of ImageJ software.

Co-inmunoprecipitation

For the immunoprecipitation of RBM10, 2 µg of anti-V5 antibody (#46-0705, Invitrogen,) was pre-coupled with 25 µL of Dynabeads Protein G (#10004D, Thermo Scientific) in 250 µL of RIPA buffer with protease inhibitors for 1 hour at RT in a rotating wheel. Then, 500 µg of protein extracted with RIPA buffer from NCIH1944 cells overexpressing RBM10 was added to the beads and incubated overnight at 4 ºC at 15 rpm. As a negative control, a mouse antibody against immunoglobulin G (IgG) was used (#12-371, Merck Millipore). Next day, beads were washed with RIPA buffer + protein inhibitors and three times with PBS + protein inhibitors for 15 min in the rotating wheel at 4 ºC and eluted in 4× loading buffer for western blotting (4% SDS, 0.2% bromophenol blue, 20% glycerol, 200 mM DTT and 0.1M Tris HCl pH 6.8). Then, interaction between RBM10 and SF3B1, SF3B2 or DHX15 was monitored by inmublotting loading the inmunoprecipitated samples and 10% of total protein as inputs. Antibodies are specified in Table S2.

IP-MS/MS

The IP for MS/MS analysis was performed essentially as described in the previous section but in native instead denaturing conditions. To this extent IP buffer (20mM tris Hcl pH 8, 140 mM KCl, 1.8 mM MgCl_2_, 0,2% IGEPAL, 1mM DTT, 40 U/mL SUPERase·In™ RNase Inhibitor, 1mM PMSF, 0.2 mg/mL heparin and protein inhibitors), washing buffer (20mM tris Hcl pH 7.5, 140 mM KCl, 1.8 mM MgCl_2_, 0,2% IGEPAL and 1mM DTT) and blocking buffer (20mM tris Hcl pH 7.5, 140 mM KCl, 1.8 mM MgCl_2_, 0,2% IGEPAL, 1mM DTT, 2% BSA and 0.5 mg/mL heparin) were used instead RIPA. 800 µg of protein were used per IP. Samples were prepared in triplicates.

Proteomic analysis was performed by the Centre for Cooperative Research in Biosciences (CICbiogune) using a nanoscale liquid chromatography tandem mass spectrometry (nLC MS/MS) with the instrument EVOSEP ONE (nLC) coupled to a TIMS Tof Pro (MS/MS). Triplicates were processed in parallel and label-free quantification was performed with MaxQuant software. Proteins with more than 0.05 False Discovery Rate (FDR) were filtered out. To define potential interactors, a one-side T-test was performed comparing the label free quantification (LFQ) intensity of each identified protein in IP-RBM10 samples vs IP-IgG samples. Proteins that presented more than 0.05 *p*-value were selected for further analysis. STRING (<https://string-db.org/>) was used to identify functional protein association networks.

Cell proliferation assays

To study cell viability, resazurin assays were carried out. 4,000 NCIH1944 cells or 1,000 NCIH2291 cells were seeded in 96-well plates in technical triplicates for each condition. At different time points, cells were treated with resazurin sodium salt solution (0.12 mM) (Sigma Aldrich, Merck) and incubated in darkness at 37 ºC for 4 hours. Then, 3% of SDS was added and fluorescence at 600 nm was measured in a Glomax® Discover Multimode Microplate Reader. Three biological replicates were performed for each condition.

Colony assay

To study the ability of the cells to form colonies, 4,000 NCIH1944 cells or 5,000 NCIH2291 cells were seeded in triplicates in six-well plates and incubated at 37 ºC for 14 days. Then, cells were incubated at RT with 0.1% crystal violet, 1% methanol and 1% formaldehyde solution for 20 minutes to fix and stain colonies. Plates were washed with water and colonies’ images were automatically recorded and quantified with Licor Odyssey 9120 Imaging System on the “700 channel” position. At least three biological replicates were performed for each condition and each cell line.

Patients’ cohort analysis

CPTAC-LUAD rRNA-depleted RNA-Seq aligned genomic BAM files were downloaded from GDC and converted to FASTQ with samtools v1.7. CIRCexplorer3 was used to extract and quantify circRNA expression in non-tumoral and tumoral samples. CIRCexplorer3 applies a new quantitation parameter, fragments per billion mapped bases (FPB), to evaluate circular RNA expression individually by fragments mapped to circRNA-specific back-splicing junction sites (FPBcirc).

In-house LUAD patient cohort was established from paired tumoral and adjacent non-tumoral tissues collected from 70 LUAD patients [12]. RNA was extracted and circHIPK3, lineal HIPK3 and RBM10 was measured by RT-qPCR. Expression was normalized using GAPDH as the endogenous control and compared to the average values.

Photoactivable-Ribonucleoside-Enhanced Crosslinking and Inmunoprecipitation (PAR-CLIP data analysis)

RBM10 PAR-CLIP sequencing reads were downloaded from NCBI SRA database (PRJNA192838). 3' adapter sequences were identified with FastQC and trimmed with Trim Galore (v0.6.10). Reads were aligned with bwa aln (v0.7.17) to the human reference genome (GRCh38) allowing at most two mismatches or indels. The alignments were converted into pileup files with SAMtools mpileup (v1.7) and T-to-C transitions were calculated with a custom Python script.

Cell-derived xenografts (CDXs)

To generate cell-derived xenografts mouse models of human lung adenocarcinoma tumors, H1944-EV or H1944-circHIPK3 transfected cells (2×10^6^ cells in 250 μL of 1:1 Matrigel:RPMI) were subcutaneously injected into the flanks of 2-month-old male NOD Scid Gamma (NSG) mice. At least 4 mice were injected for each condition. Tumor growth was assessed 5 weeks post-injection by measuring the size and weight of the tumors. Tumor volume was calculated using the formula: $V={\frac{1}{2}ab}^{2}$, where “a” represents the tumor’s longer axis and “b” represents the shorter axis*.*

Statistical analysis

Statistical analysis was performed using Graphpad Prism (https://www.graphpad.com/ scientific-software/prism/). For the analyses, unpaired t-tests or Mann-Whitney tests were applied and *p*-values ≤ 0.05 were considered statistically significant.

# Extended Data Figures

**
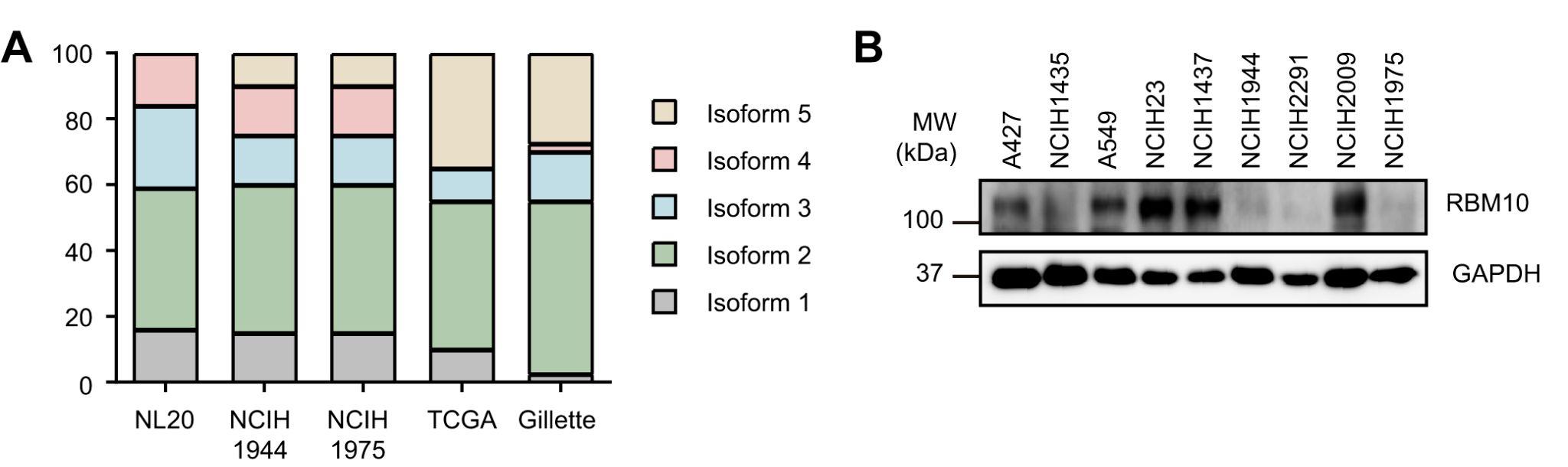
**

**Figure S1.** (**A**) Distribution of RBM10 isoforms in non-carcinogenic lung cell line NL20, two carcinogenic LUAD cell lines, NCIH1944 and NCIH1975 and 2 cohorts of patients – TCGA [13] and Gillette et al [1] (**B**) RBM10 expression monitored by immunoblot in a panel of LUAD cell lines. Actin is used as loading control.

**
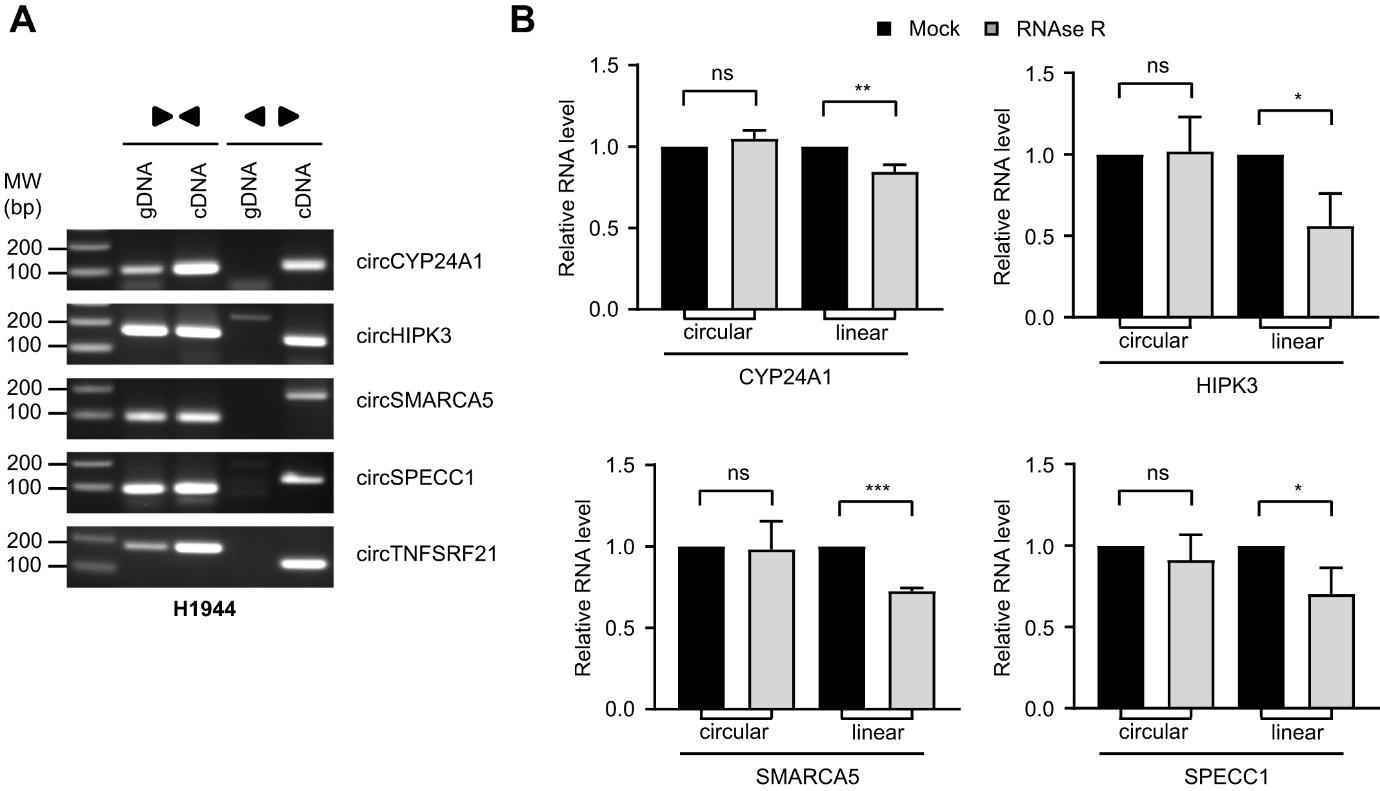
**

**Figure S2.** (**A**) CircCYP24A1, circHIPK3, circSMARCA5 circSPECC1 and circTNFSRF21 were amplified from cDNA or gDNA from NCIH1944 cells with divergent (◄►) and convergent primers (►◄), respectively and PCR products resolved on an agarose gel. (**B**) qPCR assays to determine linear or circular CYP24A1, SMARCA5, HIPK3 and SPECC1 RNA levels in NCIH1944 cells with and without RNase R treatment. * P > 0.05; ** P > 0.01; *** P > 0.001; ns: non-statistically significant; unpaired t-test.


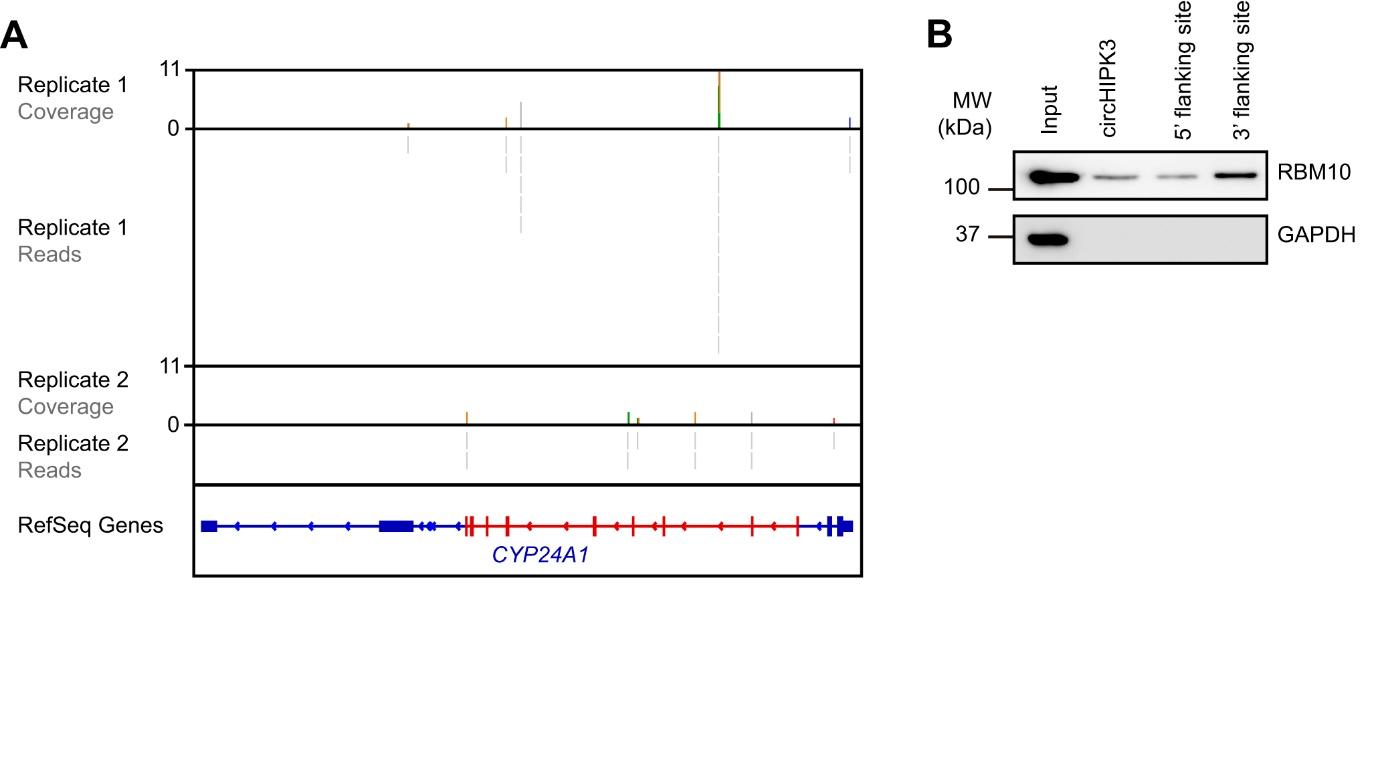


**Figure S3.** Interaction RBM10 and circRNAs. (**A**) RBM10 PAR-CLIP reads mapping CYP24A1 locus from replicate 1 (SRA ID: SRR771562) and replicate 2 (SRA ID: SRR771563) from Wang et al 2013 [14] . T-to-C transitions are marked with green (T) and orange (C) bars in the coverage tracks. circCYP24A1 (exons 3-11) is marked in red in the RefSeq Genes track. (**B**) RNA–protein complexes formed between biotin-labeled circHIPK3 and 5’/3’ flanking sites and extracts of NCIH1944 expressing RBM10 were purified on streptavidin magnetic beads and monitored for the presence of V5-RBM10 by immunoblot analysis with anti-V5 (Sigma). Biotin-labeled sequences (lanes 2 to 4) were incubated with NCIH1944 extract (input, lane 1).


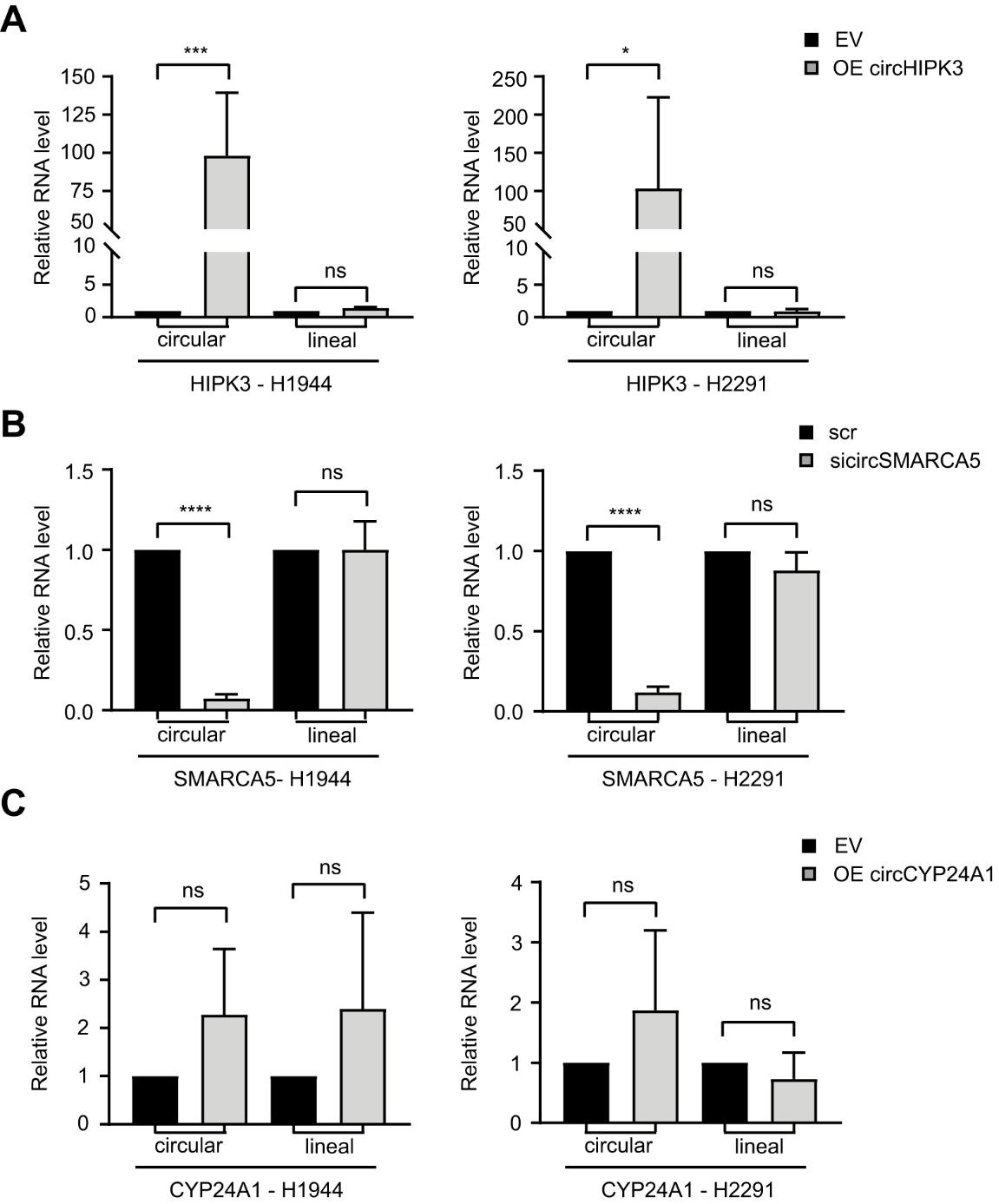


**Figure S4.** Analysis of expression levels of circRNAs upon circRNA modification. Relative expression levels of HIPK3 upon circHIPK3 ectopic expression (**A**) or SMARCA5 upon circSMARCA5 silencing (**B**) for 48 hours in NCIH1944 (left) and H2291 (right). EV was used as control. * P > 0.05; *** P > 0.001; **** P > 0.0001; ns: non-statistically significant; unpaired t-test.


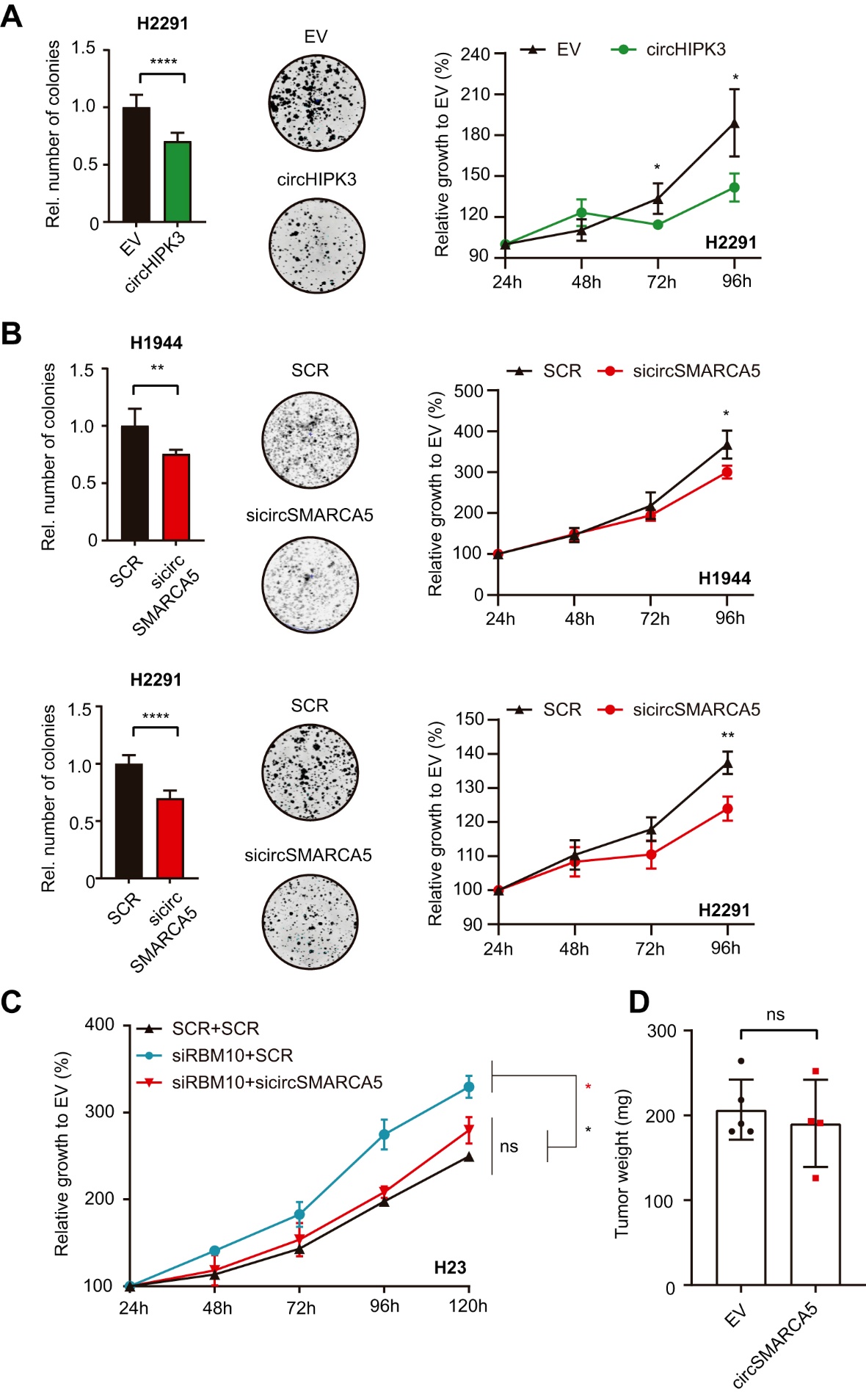


**Figure S5.** (**A**) Clonogenic and viability assays after circHIPK3 ectopic expression in NCIH2291 (* *P* < 0.05, unpaired t-test). (**B**) Clonogenic and viability assays after circSMARCA5 silencing in NCIH1944 and NCIH2291. (**C**) Rescue experiment in NCIH23 WT cells under RBM10 silencing and subsequent modulation of circSMARCA5 (* P < 0.05; ns: non- significant; unpaired t-test at 120h). (**D**) Tumor weight quantification of *ex vivo* tumors of NCIH1944-sicircSMARCA5 or EV cell-derived xenografts.

**
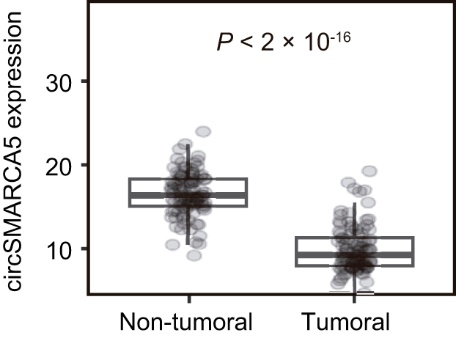
**

**Figure S6.** Box plot comparing circSMARCA5 expression between non-tumoral and tumoral samples from the CPTAC (Mann-Whitney test).


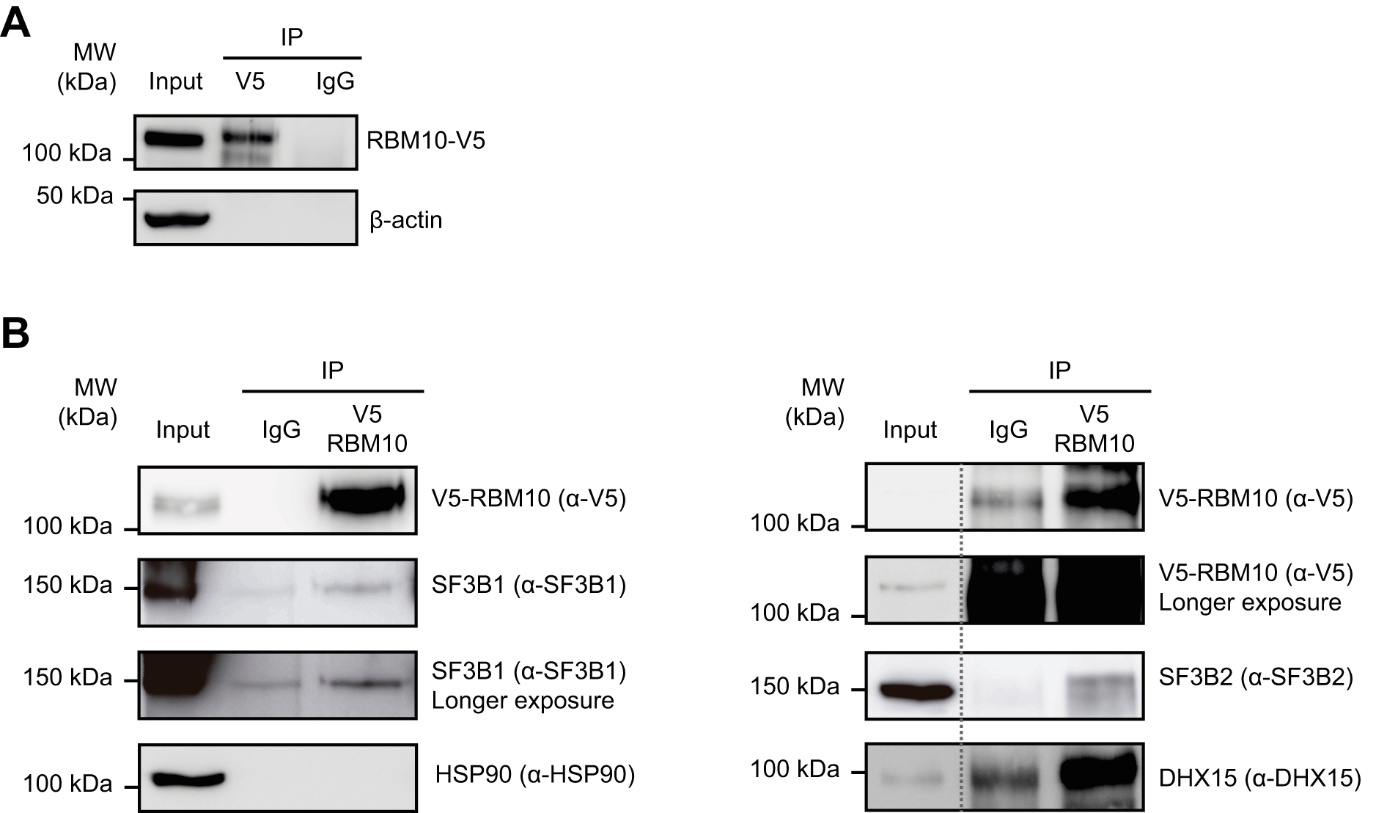


**Figure S7.** (**A**) Immunoblot monitoring the immunoprecipitation of V5-RBM10 using an anti-V5 antibody, and IgG as control. GAPDH was used as a negative control for non-specific binding. (**B**) Immunoblot assays to confirm the physical interaction between RBM10 and SF3B1, SF3B2 and DHX15. HSP90 was used as a negative control for non-specific binding. The dashed lines in the Western blots indicate that the image has been cropped to present the relevant bands more concisely. Samples originate from the same experiment.


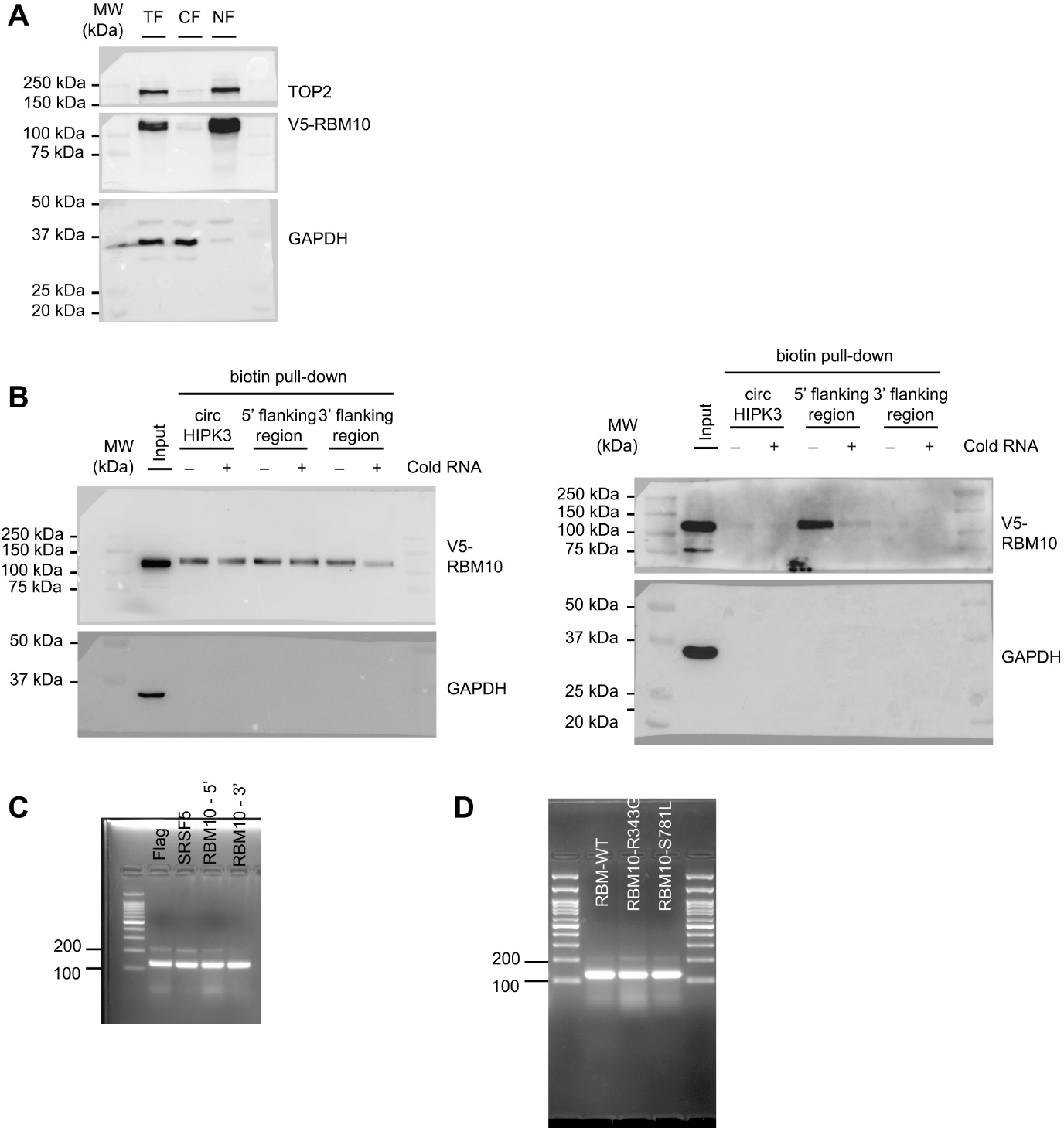


**Figure S8.** Original uncropped blots and gels of the main figures. (**A**) Uncropped blots used for preparation of Fig. 1D (**B**) Uncropped blots used for preparation of Fig. 1F (**C**) Uncropped agarose gels used for preparation of Fig. 1G. (**D**) Uncropped agarose gels used for preparation of Fig. 1H.

**
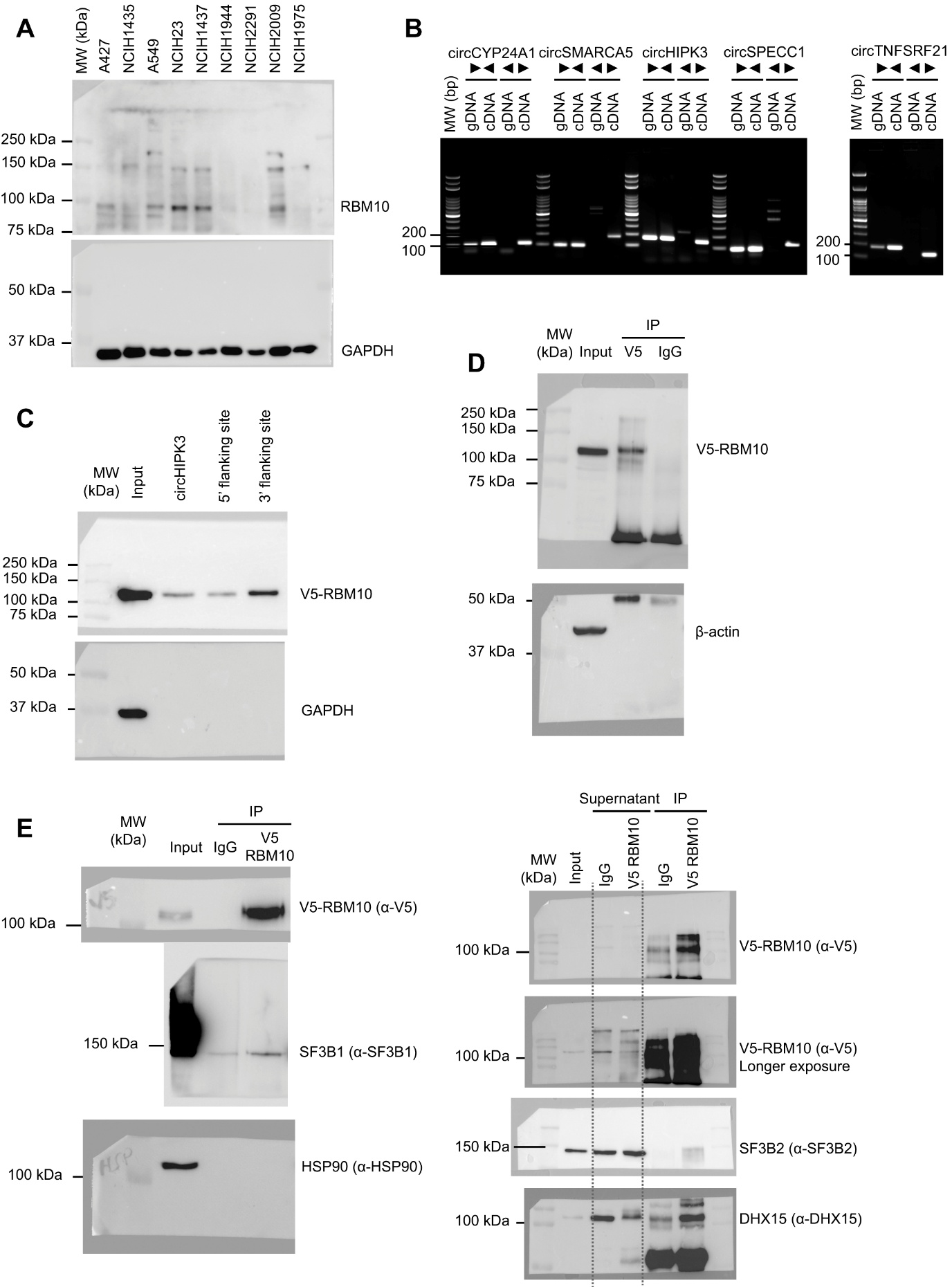
**

**Figure S9.** Original uncropped blots and gels of the supplementary figures. (**A**) Uncropped blots used for preparation of Fig. S1B. (**B**) Uncropped agarose gels used for preparation of Fig. S2A. (**C**) Uncropped blots used for preparation of Fig. S3B. (**D**) Uncropped blots used for preparation of Fig. S7A. (**E**) Uncropped blots used for preparation of Fig. S7B. Dashed lines delimit lanes not displayed in the figure S7B that correspond to the supernatant samples of the corresponding IP experiments.

# Extended Data Tables

**Table S1**. Top 10 circRNAs detected upon RBM10 restoration. Columns indicate circRNA name; Average FC_RBM10/EV_, calculated average FC_RBM10/EV_; Average number of reads, detected across all replicates in all samples. Upregulated or downregulated genes in both replicates are marked with '+'. circRNAs labeled in red indicate those circRNAs selected for validation.

| circRNA | Average  FC RBM10/EV | Average number of reads | Upregulated | Downregulated |  |
| --- | --- | --- | --- | --- | --- |
|  |  |  |  |  |  |
| CYP24A1 | 1.69 | 930.25 | + |  |  |
| SPECC1 | 1.64 | 32.5 | + |  |  |
| HIPK3 | 1.25 | 59.25 | + |  |  |
| DNAH14 | 1.37 | 30.25 | + |  |  |
| NEIL3 | 1.35 | 10.00 | + |  |  |
| RNF13 | 0.76 | 34.25 |  | + |  |
| SMARCA5 | 0.52 | 33.25 |  | + |  |
| TNFRSF21 | 0.51 | 30.75 |  | + |  |
| CEP192 | 0.76 | 24.50 |  | + |  |
| ARHGAP12 | 0.65 | 23.00 |  | + |  |

**Table S2**. List of antibodies used in this study.

| Antibody | Dilution | Source | Identifier |
| --- | --- | --- | --- |
| RBM10 (H-4) | 1:150 | Santa Cruz Biotechnology | sc-515548 |
| Anti-V5 antibody | 1:3000 | Invitrogen | 46-0705 |
| Anti-TOP2A | 1:500 | Sigma Life Science | HPA006458 |
| Anti-SF3B1 (D7L5T) | 1:1000 | Cell Signaling | #14434 |
| Anti-SF3B2/SAP-145 (C-12) | 1:1000 | Santa Cruz Biotechnology | sc-514930 |
| Anti-DDX15 | 1:1000 | Santa Cruz Biotechnology | sc-271686 |
| Anti-HSP90 | 1:1000 | Cell Signalling | 4877S |
| Anti-β-actin monoclonal antibody | 1:20000 | Sigma Life Science | A5441 |
| GAPDH (0411) | 1:1000 | Santa Cruz Biotechnology | sc-47724 |
| Polyclonal Goat Anti-mouse Immunoglobulins/HRP | 1:1000 | Dako | P0447 |
| Polyclonal Goat Anti-rabbit Immunoglobulins/HRP | 1:2000 | Dako | P0448 |

# Supplementary File Descriptions

**File S1**. circRNAs detected upon RBM10 restoration. Columns indicate circRNA, name and coordinates; Parental gene, corresponding parental gene; number of reads detected in each control and RBM10 replicates; FC_RBM10/EV_, calculated for each replicate and the average; average number of reads detected across all replicates in all samples. Upregulated or downregulated genes in both replicates are marked with '+'.

**File S2.** List of oligonucleotides used in this study. (**S2.1**) Oligonucleotides for RBM10 isoforms analysis; (**S2.2**) Oligonucleotides for RT-qPCR; (**S2.3**) Oligonucleotides for RNA *in vitro* synthesis; (**S2.4**) Divergent and convergent primers for figure S2.a.

# References

1. Gillette MA, Satpathy S, Cao S, Dhanasekaran SM, Vasaikar S V., Krug K, et al. Proteogenomic Characterization Reveals Therapeutic Vulnerabilities in Lung Adenocarcinoma. Cell. 2020;182:200-225.e35.
2. Tang Z, Kang B, Li C, Chen T, Zhang Z. GEPIA2: an enhanced web server for large-scale expression profiling and interactive analysis. Nucleic Acids Res. 2019;47:W556–60.
3. Vaquero-Garcia J, Barrera A, Gazzara MR, González-Vallinas J, Lahens NF, Hogenesch JB, et al. A new view of transcriptome complexity and regulation through the lens of local splicing variations. Elife. 2016;5.
4. Rosenbluh J, Mercer J, Shrestha Y, Oliver R, Tamayo P, Doench JG, et al. Genetic and Proteomic Interrogation of Lower Confidence Candidate Genes Reveals Signaling Networks in β-Catenin-Active Cancers. Cell Syst. 2016;3:302-316.e4.
5. Liang D, Wilusz JE. Short intronic repeat sequences facilitate circular RNA production. Genes Dev. 2014;28:2233–47.
6. Zhang H, Meng F, Dong S. circSMARCA5 Promoted Osteosarcoma Cell Proliferation, Adhesion, Migration, and Invasion through a Competing Endogenous RNA Network. Biomed Res Int. 2020;2020:1–8.
7. Guo Y, Wang X, Du Y, Zhao Y, Gao L, Hao Y, et al. The splicing factor SF3B1 confers ferroptosis resistance and promotes lung adenocarcinoma progression via upregulation of SLC7A11. Cancer Gene Ther. 2024;31(10):1498-1510.
8. Zhang X-O, Dong R, Zhang Y, Zhang J-L, Luo Z, Zhang J, et al. Diverse alternative back-splicing and alternative splicing landscape of circular RNAs. Genome Res. 2016;26:1277–87.
9. Beringer M, Pisano P, Di Carlo V, Blanco E, Chammas P, Vizán P, et al. EPOP Functionally Links Elongin and Polycomb in Pluripotent Stem Cells. Mol Cell. 2016;64:645–58.
10. Schmok, JC, Jain, M, Street, LA, Tankka AT, Schafer D, Her H-L et al. Large-scale evaluation of the ability of RNA-binding proteins to activate exon inclusion. Nat Biotechnol. 2024; 42:1429–1441.
11. Bradford M. A Rapid and Sensitive Method for the Quantitation of Microgram Quantities of Protein Utilizing the Principle of Protein-Dye Binding. Anal Biochem. 1976;72:248–54.
12. Peinado, P., Andrades, A., Cuadros, M., Rodriguez, M. I., Coira, I. F., Garcia, D. J., et al. Multi-omic alterations of the SWI/SNF complex define a clinical subgroup in lung adenocarcinoma. Clinical epigenetics. 2022; 14(1), 42.
13. Campbell JD, Alexandrov A, Kim J, *et al.* Distinct patterns of somatic genome alterations in lung adenocarcinomas and squamous cell carcinomas. *Nat Genet* 2016; **48**:607–616.
14. Wang Y, Gogol‐Döring A, Hu H, *et al.* Integrative analysis revealed the molecular mechanism underlying RBM10‐mediated splicing regulation. *EMBO Mol Med* 2013; **5**:1431–1442.
